# Influence of sample collection tube method, anticoagulant-containing plasma versus serum, on influenza virus hemagglutination inhibition titer and microneutralization titer serological assays

**DOI:** 10.1186/s12913-018-3465-3

**Published:** 2018-08-22

**Authors:** Brian J. Morrison, Nicholas J. Martin, Tauseef Rehman, Dan Ewing, Robin L. Dewar, Julia Metcalf, Peifang Sun, John Beigel, Thomas C. Luke, Kanakatte Raviprakash

**Affiliations:** 1 Viral and Rickettsial Diseases Department, Infectious Diseases Directorate, Naval Medical Research Center, 503 Robert Grant Avenue, Silver Spring, MD 20910 USA; 2 Naval Medical Research Center-Asia, Singapore, Singapore; 3 Leidos Biomedical Research, Frederick, MD 21702 USA; 4 National Institute of Allergy and Infectious Diseases, National Institutes of Health, Bethesda, MD 20817 USA

**Keywords:** Influenza virus, Hemagglutination inhibition titer, Serum, Plasma, Microneutralization titer, Anticoagulants, Collection tubes, Serology, Variability

## Abstract

**Background:**

The hemagglutination-inhibition (HAI) assay is a critical component for measurement of immunogenicity in influenza vaccine development. It is unknown if the results can be influenced by sample type and anticoagulants. The purpose of this study was to evaluate the influence of different sample collection methods, in particular different anticoagulants, and choice of plasma or serum, on influenza virus serological assays.

**Methods:**

Blood samples from thirty donors previously immunized against influenza viruses were collected using six different types of blood collection tubes, two of which collect serum and four of which contain various anticoagulants for collecting plasma. Serum: (1) serum separator tubes (SST); and (2) Plus Plastic serum “red-top serum” tubes. Plasma: (3) spray-coated K2 ethylenediaminetetraacetic acid (EDTA) tubes: (4) Sodium Heparin tubes; (5) Citrate tubes with 3.2% sodium citrate solution; and (6) Glass Blood Collection tubes with acid citrate dextrose. Samples were tested against three different influenza viruses (A/California/07/2009 (H1N1pdm09), A/Texas/50/2012 (H3N2), and B/Massachusetts/2/2012) for hemagglutination inhibition titer and virus neutralization titer via a microneutralization (MN) assay, and data compared to that obtained for standard serum sample collected in SST.

**Results:**

HAI and MN titers against type A viruses were within two dilutions compared to SST collection method over 96% of the time irrespective of sample type or anticoagulant. However, HAI titers for type B virus were more variable across different collection methods. EDTA plasma samples were greater than two dilutions higher than SST serum samples 70% (21 of 30 samples) of the time. In contrast, MN titers were within two dilutions over 96% of the time, with the highest deviation noted in acid citrate dextrose plasma samples (3 of 30 samples tested, 10%).

**Conclusions:**

These data provide useful guidelines for sample collection and serology testing when screening: (i) influenza vaccine immunogenicity antibody response; (ii) antibody responses to newly emerging viral strains; and (iii) clinical samples for anti-influenza antibody activity.

**Electronic supplementary material:**

The online version of this article (10.1186/s12913-018-3465-3) contains supplementary material, which is available to authorized users.

## Background

Globally influenza remains a significant cause of morbidity/mortality in human populations [1], with 3–5 million severe clinical infections yearly resulting in approximately 250,000–500,000 fatalities [2, 3]. Antibodies (Abs) that are specific to the viral envelope protein hemagglutinin (HA) prevent binding of virus to target cell sialic acid residues and are largely thought to mediate immune protection against influenza viruses [4]. Viral HA will cross link erythrocytes, leading to increased rates of sedimentation. Hemagglutination-inhibition (HAI) assays measure antibody titers, by measuring the inhibition of the agglutination of erythrocytes [5, 6]. An additional serological assay that is employed to assess influenza Abs includes the microneutralization (MN) assay, with an enzyme-linked immunosorbent assay (ELISA)-based endpoint assessment, that is used to detect neutralizing Ab titers through inhibition of virus infection [7, 8]. Although a variety of additional serological assays to assess functional anti-influenza antibody titers exist [9–11], the MN and, in particular, the HAI assays remain gold-standard assays to evaluate immunogenicity of influenza vaccines and to measure serological responses to natural infection [12]. However, there is increasing awareness of the variability of influenza serological assay results [12] and the need to improve inter-laboratory agreement on serological assay standards [13]. This is especially relevant for: (i) assessment of influenza vaccine immunogenicity; (ii) epidemiological studies seeking to catalog newly emerging viral strains in an affected region over time; and (iii) assessment of clinical samples for anti-influenza Ab activity.

Developing effective vaccines against seasonal and pandemic influenza is a public health priority. Variation across laboratories, including operator inexperience influencing reproducibility and lack of common standardized neutralization and HAI assays, in serological procedures limits comparisons of vaccine strategies and vaccine efficacy [12, 14]. Protocols are also not standardized in regards to expression of endpoint and this contributes to variable reporting [14, 15]. An additional variable that can affect serological results is the choice of sera versus plasma for measuring samples, and the presence of anticoagulants in plasma.

Historically, serum has been the sample of choice for HAI assessments [16, 17]. However, plasma has been gaining popularity for human subject research studies [18]. Plasma has the potential disadvantage compared to sera in diagnostic assays, as anticoagulants have been demonstrated to interfere with antibody-antigen interaction and some enzyme reagents [19, 20]. However, in regards to the HAI assay, we have previously determined that both serum and plasma (serum-citrate plasma and serum-heparinized plasma) can be used in serodiagnostic assays (seroconversion rates remaining unaffected by sample type), but that the use of plasma samples may underestimate HAI titers [21]. Reasons that anticoagulants may affect assay readout are varied but include: (i) sodium citrate or EDTA acting as chelating agents and binding enzyme cofactors affecting enzyme activity in assays (increasing or decreasing titers); (ii) heparin being shown to interfere with antibody-antigen reactions [19, 20] potentially biasing towards decreased titers; and (iii) plasma clots mimicking agglutination patterns in affected wells biasing towards a lower titer readout. A more detailed analysis of the influence of sample type (serum versus plasma) and the sample collection method (including various anticoagulant agents) on HAI and MN assays is warranted as plasma becomes a more routinely obtained specimen. This is especially relevant as sample collection methods may either overestimate or underestimate seroprevalence rates and this can impact assessment of vaccine efficacy, as new influenza vaccine are expected to meet specific seropositive and seroconversion rate thresholds, such as those outlined by the United States Food and Drug Administration (FDA) guidelines on licensure [22].

The purpose of this study was to evaluate the influence of sample collection methods on influenza virus serological assays to inform on guidelines for sample collection and assay standardization. In particular, we assessed the effect of the use of various collection tubes and anticoagulants on the HAI and MN variability compared to standard serum samples collected using serum separator tubes. Blood samples collected from thirty donors previously vaccinated against influenza were collected using six different sample collection tubes (Serum: (1) serum separator tubes (SST); and (2) Plus Plastic serum “red-top serum” tubes. Plasma: (3) spray-coated K2 ethylenediaminetetraacetic acid (EDTA) tubes: (4) Sodium Heparin tubes; (5) Citrate tubes with 3.2% sodium citrate solution; and (6) Glass Blood Collection tubes with acid citrate dextrose (ACD) and serum or plasma samples were prepared. Three influenza virus strains were used in this study to evaluate potential differences in HAI and MN titer values associated with these variables.

## Methods

### Ethics statement

The samples obtained were part of an observational study conducted at the National Cancer Institute (NCI) Frederick Cancer Research and Development Center. The study protocol and consent were approved by the NCI Institutional Review Board (Protocol OH99-N046). ClinicalTrials.gov Identifier: NCT00339911.

### Viruses

A/California/07/2009 (H1N1pdm09), A/Texas/50/2012 (H3N2), and B/Massachusetts/2/2012 viruses (International Reagent Resource; Manassas, VA) were amplified on fertilized chicken eggs (Charles River Laboratories, Wilmington, MA). Eggs were incubated at 37 °C with humidity and 0% CO_2_ for 48 h, before transfer to 4 °C for an additional 24 h. Allantoic fluid was then collected, spun down to remove debris, and initially titered with turkey red blood cells (Lampire Biological Laboratories; Pipersville, PA). Of note, B/Massachusetts/2/2012 was not ether split prior to conducting serological assays.

### Samples

Blood samples were collected from thirty healthy donors previously immunized against influenza viruses using six different collection tubes and sera or plasma were prepared according to the manufacturers’ instructions. Serum: (1) Vacutainer® serum separator tubes (SST), (Becton Dickinson and Company (BD), Franklin Lakes, NJ); and (2) Vacutainer® Plus Plastic serum “red-top serum” (RT) tubes (BD). Plasma: (3) Vacutainer® spray-coated K2 ethylenediaminetetraacetic acid (EDTA) tubes, 7.2 mg (BD): (4) Vacutainer® Sodium Heparin (Hep plasma) tubes (BD, #36781) containing sodium heparin 95 USP; ≥180 USP units/mg; (5) Vacutainer® Citrate (Cit plasma) tubes (BD) containing 0.5 mL of citric acid (8.0 g/L) solution; and (6) Vacutainer® Glass Blood Collection tubes with acid citrate dextrose (ACD) (BD, #34606) containing trisodium citrate (22.0 g/L), citric acid (8.0 g/L) and dextrose (24.5 g/L).

### HAI assay

The samples were screened for anti-HA influenza antibody titers against the A/California/07/2009 (H1N1pdm09), A/Texas/50/2012 (H3N2), and B/Massachusetts/2/2012 viruses using an assay based on the World Health Organization/Centers for Disease Control and Prevention HAI Assay [21, 23, 24]. Specific methods followed for conducting the HAI have been described elsewhere [21].

### MN assay

The MN assay used was based on the WHO protocol as previously described [25]. Briefly, the presence of serum antibodies to the hemagglutinin protein will result in inhibition of infection of Madin-Darby canine kidney (MDCK) cells (American Type Culture Collection, CCL-34; Manassas, VA) by virus. The assay was performed in two stages consisting of: (i) a virus-antibody reaction step, in which a certain amount of virus was mixed and incubated with serially diluted serum; and (ii) an inoculation step in which the mixture was inoculated into the appropriate host system – MDCK cells. The absence of infectivity constitutes a positive neutralization reaction and indicates the presence of virus-specific neutralizing antibodies in the sera. For the virus-antibody reaction step; two-fold serial dilution of serum/plasma was performed across the rows of a flat bottom 96 well plate (BD). Virus was then added to wells (diluted to 100 50% tissue culture infective dose ((TCID_50_)). Plates were then incubated for 1 h at 37 °C. MDCK cells were then added to the plate at 2.5 × 10^4^ cells/well. MDCK cell cultures were grown in Minimal Essential Medium w/ Earles salts and L-glutamine (Gibco; Langley, OK) with 5% heat-inactivated fetal bovine serum (FBS) (Quality Biological; Gaithersburg, MD) and 1% penicillin/streptomycin (P/S) (Corning; Corning, NY). For infection, virus was diluted in same media supplemented with tosyl phenylalanyl chloromethyl ketone (TPCK) (Accurate Chemical; Westbury, NY) at 2 μg TPCK/1 mL media. Prior to ELISA, cells were fixed with 80% acetone. For the ELISA procedure, mouse anti-NP mouse influenza A (from Centers for Disease Control and Prevention, #90–0026) primary antibody were diluted 1:5000 in blocking buffer (100 mL PBS, 5 g Skim milk, and 0.3 mL Tween-20). Plates were washed three times, and incubated with the primary antibody for 1 h at37°C. Secondary antibody, goat anti-mouse IgG HRP (Kirkegaard and Perry Laboratories (KPL), Gaithersburg, MD) was diluted 1:5000 in blocking buffer. Plates were washed three times, and incubated with the secondary antibody for 1 h at37°C. Plates were then washed 6 times, and incubated for 15 min in the dark at room temperature with substrate solution (KPL). 1 N sulfuric acid stop solution was added to each well and absorbance was read at 450 nm. The methodology combines culture and antigen detection by ELISA and estimate 50% reduction of viral antigen as the end point. Virus neutralization antibody endpoints are determined for each serum/plasma sample as follows: ((Average of virus control wells) + Average of negative control wells)) / 2.

### Statistical analysis

Statistics were performed using GraphPad Prism software (GraphPad; La Jolla, CA). The agreement, including bias and 95% confidence interval, between matched SST titer values and the titer values of other collection methods was assessed using a method described by Bland and Altman [26]. The geometric mean titer (GMT) of the SST value and the value of the other collection method assessed was plotted on the X-axis. The dilution factor difference between the two values was plotted on the Y-axis. Cut-off values of 2 and − 2 are plotted. A t-test relative to a threshold (TREAT) analysis [27], with a threshold set to two dilution factors equating to a four-fold or greater difference in titer (either positive or negative), was conducted to assess significance.

## Results

### HAI and MN titers for standard serum samples collected using SST

HAI and MN titer values were assessed for all thirty matched samples collected using six different collection tubes (see Additional file 1 for raw values). SST values for HAI and MN titers were used as a reference for comparison to other collection methods. Table 1 shows the frequency distribution of HAI titers against influenza A subtypes H1N1, H1N3, and influenza B for SST for all thirty samples. Table 2 shows the frequency distribution of MN titers against influenza A subtype H1N1 and influenza B for SST for all thirty samples. For influenza B virus 23/30 (70%) samples demonstrated HAI titer < 80, whereas 4/30 (13%) demonstrated MN titer < 80.

**Table 1:** Frequency distribution of HAI titers for SST

SST Titer	Type A, H1N1	Type A, H3N2	Type B
< 40	3	1	12
40 - < 80	6	5	11
80 - < 160	7	6	4
160 - < 320	9	9	3
320 - < 640	1	4	0
640 - < 1280	4	4	0
1280	0	1	0

**Table 2:** Frequency distribution of MN titers for SST

SST Titer	Type A, H1N1	Type B
< 40	2	1
40 - < 80	2	3
80 - < 160	5	3
160 - < 320	7	6
320 - < 640	9	5
640 - < 1280	4	6
1280	1	6

### Influenza B HAI titers for plasma samples containing EDTA are exaggerated

The percentage of samples recording ≥ two-dilutions variation in HAI titers for each collection method compared to the reference SST collection method is shown in Table 3. For influenza A, all samples of ACD plasma, Hep plasma, and EDTA plasma provided were within two-dilutions variation. There was one sample (1/30 (3%)) collected in RT sera and Cit plasma collection methods that was ≥ two-dilutions compared to SST sample.

**Table 3:** Number and percent samples recording ≥ two-dilution variation in HAI titer compared to SST results

Virus	RT Sera	ACD Plasma	Hep Plasma	Cit Plasma	EDTA Plasma
Type A, H1N1	1 (3.3%) (lower)	0 (0%)	0 (0%)	1 (3.3%) (lower)	0 (0%)
Type A, H3N2	0 (0%)	0 (0%)	0 (0%)	1 (3.3%) (lower)	0 (0%)
Type B	2 (6.66%) (lower)	1 (3.3%) (lower)	2 (6.66%) (1 lower, 1 higher)	2 (6.66%) (lower)	21 (70%) (higher)

The greatest variation in HAI titers compared to SST collection was for influenza B. Six samples (6/30, 20%) demonstrated a lower titer than matched SST that was ≥ two-dilutions change. A higher titer compared to SST that was ≥ two-dilutions change was noted for one (1/30 (3%)) Hep plasma sample. Additionally, 21 out of 30 (70%) EDTA plasma samples were ≥ two-dilutions higher compared to SST, with two samples (7%) being four dilutions different to matched SST titers.

**Fig. 1:**
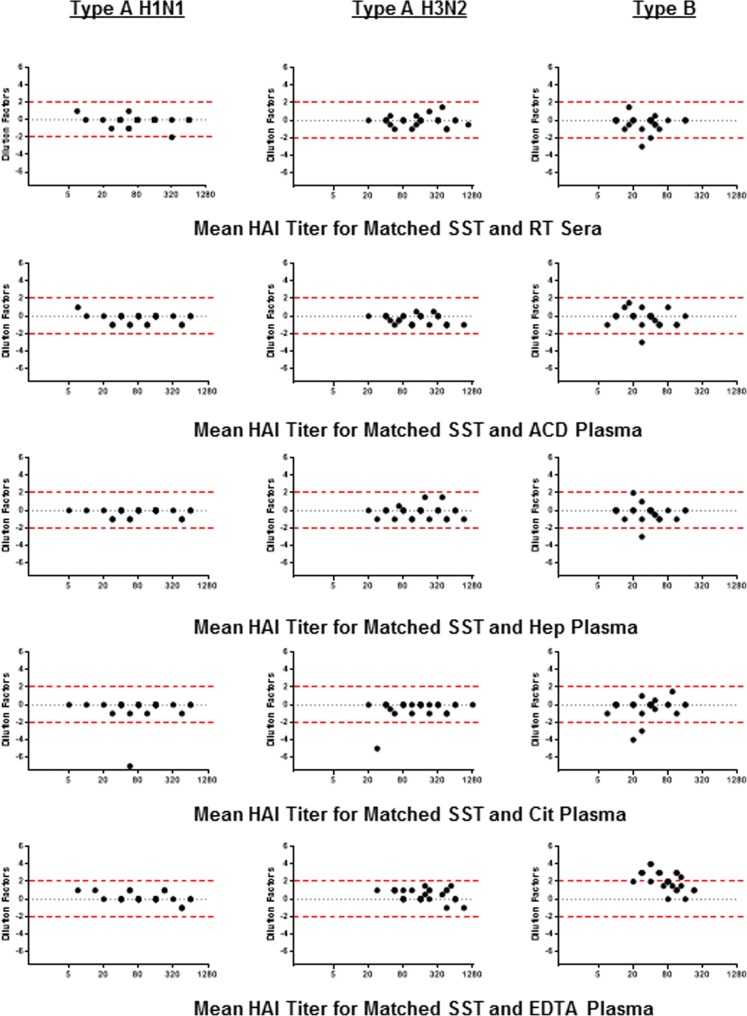
Bland-Altman plots for matched samples comparing HAI titers for SST compared to each collection method. The GMT of the SST value and the value of the other collection method assessed is plotted on the X-axis. X-axis is Log2. The dilution factor difference between the two values is plotted on the Y-axis. Cut-off values of 2 and − 2 are plotted. Note each plot shows 30 points

In order to demonstrate the variation in HAI titer values for the various collection methods compared to SST, Bland-Altman plots are graphically presented in Fig. 1 with cut-off values marked at two-dilutions factor differences. Bias and 95% confidence intervals are summarized in Table 4. HAI results for influenza B using EDTA plasma tubes were the only values where the bias dilution factor value for all samples was ≥ two dilutions different compared to SST (2.17 ± 1 standard deviation dilution factor difference). Using a TREAT analysis [27], with a threshold set to 2, we determined that the only significant deviation from SST was for EDTA plasma tubes when conducting HAI for type B influenza (*P* value < 0.0001).

**Table 4:** Bland-Altman analysis for bias and 95% confidence interval compared to SST for HAI titers

Collection Method	Bias, 95% Confidence		
	Type A, H1N1	Type A, H3N2	Type B
RT Sera	−0.1 (−1.17, 0.97)	− 0.07 (− 1.12, 0.99)	−0.25 (− 1.77, 1.27)
ACD Plasma	− 0.23 (− 1.22, 0.75)	−0.38 (− 1.4, 0.64)	−0.2 (− 1.84, 1.44)
Hep Plasma	− 0.2 (− 1, 0.6)	−0.15 (− 1.42, 1.12)	−0.2 (− 1.76, 1.36)
Cit Plasma	− 0.4 (− 2.95, 2.15)	−0.35 (− 2.23, 1.53)	−0.25 (− 2.24, 1.74)
EDTA Plasma	0.13 (− 0.86, 1.13)	0.43 (− 0.85, 1.71)	2.17 (0.17, 4.17)

Dilution factor bias for each sample collection method compared to SST is shown. Negative values indicate overall lower titer values and positive values indicate overall higher titer values compared to SST. The closer the value is to zero the better the agreement between the two collection tube methods. The 95% confidence interval is also shown demonstrating the dilution factor limits of agreement between the two methods

### MN titers are more uniform than HAI titers across sample types for both type A and type B viruses

The percentage of samples recording ≥ two-dilution change in MN titers for each collection method compared to the reference SST collection method is shown in Table 5. For influenza A, 1/30 (3%) had H1N1 MN titers higher than matched SST samples by ≥ two-dilution change. For influenza B, values were more varied compared to SST collection. Samples that were ≥ two-dilutions different compared to SST included 1/30 (3%) for RT sera and Cit plasma, and 3/30 (10%) for ACD plasma.

**Table 5:** Number and percent samples recording ≥ two-dilution variation in MN titer compared to SST results

Virus	RT Sera	ACD Plasma	Hep Plasma	Cit Plasma	EDTA Plasma
Type A, H1N1	0 (0%)	0 (0%)	1 (3.3%) (higher)	0 (0%)	0 (0%)
Type B	1 (3.3%) (higher)	3 (10%) (1 lower, 2 higher)	0 (0%)	1 (3.3%) (lower)	0 (0%)

**Fig. 2:**
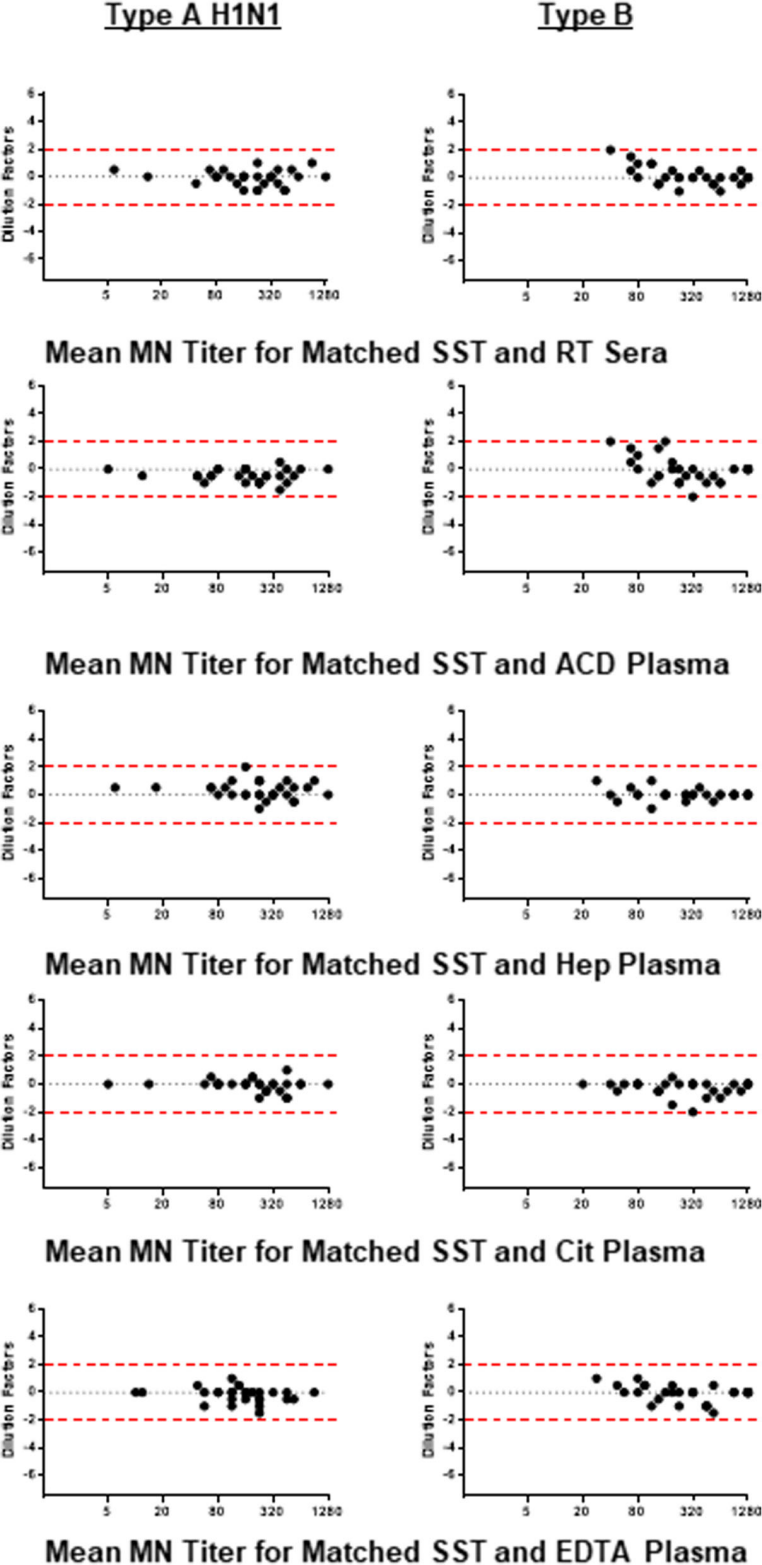
Bland-Altman plots for matched samples comparing MN titers for SST compared to each collection method. The GMT of the SST value and the value of the other collection method assessed is plotted on the X-axis. X-axis is Log2. The dilution factor difference between the two values is plotted on the Y-axis. Cut-off values of 2 and − 2 are plotted. Note each plot shows 30 points

MN titer values for each collection method compared to SST are graphically presented in Fig. 2 and the analysis is summarized in Table 6. Although 10% of samples collected using the ACD plasma collection method deviated ≥ two-dilutions different to SST collection the average bias (− 0.05 ± 0.9 standard deviation dilution factor difference) did not pass the threshold requirement of 2. Using a TREAT analysis, with a threshold set to 2, we determined that the average fold change values for MN collected with ACD plasma was not significantly different to SST.

**Table 6:** Bland-Altman analysis for bias and 95% confidence interval compared to SST for MN titers

Collection Method	Bias, 95% Confidence	
	Type A, H1N1	Type B
RT Sera	−0.08 (−1.18, 1.01)	0.13 (− 1.18, 1.44)
ACD Plasma	−0.45 (−1.35, 0.46)	− 0.05 (− 1.88, 1.78)
Hep Plasma	0.32 (− 0.88, 1.51)	0.02 (− 0.73, 0.77)
Cit Plasma	− 0.08 (− 0.94, 0.77)	−0.28 (− 1.3, 0.74)
EDTA Plasma	− 0.17 (− 1.17, 0.84)	−0.08 (− 1.27, 1.1)

Dilution factor bias for each sample collection method compared to SST is shown. Negative values indicate overall lower titer values and positive values indicate overall higher titer values compared to SST. The closer the value is to zero the better the agreement between the two collection tube methods. The 95% confidence interval is also shown demonstrating the dilution factor limits of agreement between the two methods

## Discussion

The serological identification of antibodies/antigens in serum/plasma is a rapidly progressing field with utility for both clinicians and scientist. Clinically, serology is useful for: (i) diagnosis; and (ii) monitoring vaccine/treatment efficacy in order to aid clinical management decisions. Scientifically, serology is useful for: (i) conducting seroepidemiological investigations; and (ii) to inform on the breadth of host immune responses to a particular pathogen [28]. Although serological assays have improved in recent years, variability in assay methods across laboratories remains an issue impeding greater standardization in result reporting. This is particularly true in the field of influenza. It has become increasingly recognized that blood collection methods remain a frequently overlooked aspect of protocols that may be responsible for uncontrolled variability [29]. As such, we sought to assess the influence of different blood collection methods, specifically different blood tubes, for influenza HAI and MN reporting.

In general, intra-laboratory titer values that are within two dilution factors (four-fold difference) from duplicate and/or repeated testing of a particular sample is considered acceptable and the values considered to be comparable [12, 30]. In this study we compared HAI titers results from various collection tube methods to SST and demonstrated that there was no alternative sample that across strains had full concordance with the HAI obtained from a SST sample. Agreement between the test sample and SST for single strains ranged between 30 and 100%. Matched samples from the various collection methods were within the two-dilution criteria when assessed for type A and B influenza viruses and for HAI and MN 93% of the time for RT Sera, Hep plasma, and Cit plasma. EDTA plasma tested for type B influenza HAI titers demonstrated ≥ two-dilution variation in 70% of samples. Similarly, but to a lesser degree, 10% of samples from ACD plasma were different to SST for type B influenza MN titers. Based on these observations, we believe that RT serum, Hep plasma, ACD plasma, and Cit plasma could be used in place of or in addition to SST to measure HAI and MN titers if needed, though SST remains the gold standard and should still be used whenever possible.

MN assays are often reported to have greater inter- and, and importantly to the present study, intra-laboratory variability compared to HAI assays [12, 14, 31]. We did not find this to be the case in the present study. Despite difference in starting material (sera and plasma based on the collection method) we found that only 2% (6 out of 300) of samples deviated from the SST reference for the MN assay. Alternatively, we saw 6.9% (31 out 450) of samples deviate from the SST reference for the HAI assay. Deviation from the SST values occurred more frequently for influenza B assays for both MN (3.3%; 5 out of 150) and HAI (18.7%; 28 out of 150), then for influenza A assays. The greatest variation in the MN assay was for influenza B when using ACD plasma as the starting material. ACD plasma resulted in a greater than two-dilutions higher titer two times compared to SST and a lower than two-dilutions titer once compared to SST. All of the higher titer results for ACD plasma occurred when MN titers for the SST were low (titers of 10–40). At titers above 40 there was more agreement on titer amongst sample collection methods, including ACD plasma. This may indicate that ACD plasma has either greater sensitivity for influenza B MN titer assessment at lower titer than SST or that ACD plasma overestimates titers.

For the HAI assay the most interesting finding was the observation that the EDTA plasma collection method resulted in a ≥ two-dilutions change compared to SST in 21 out of 30 samples assessed for influenza B. Additionally, in no cases was the influenza B HAI titer result for EDTA plasma lower than for SST. The observation that titers for influenza B HAI were higher for EDTA plasma collection tubes compared to SST might indicate that they are potentially overestimating the real titer. It should be noted that the SST titers for influenza B were routinely low, ranging from 10 to 160, and this may have influenced variation in the sample set.

Assessment of antibody responses to specific influenza subtypes is an important diagnostic, epidemiological, and immunological tool [32]. The observation that we saw higher HAI titers for EDTA plasma for influenza B compared to SST has implications related to seroprevalence and seroconversion studies. Overestimating and underestimating the seroprevalence rates in response to a newly emerging influenza virus is of concern. Additionally, as new influenza vaccines are expected to meet specific seropositive and seroconversion rate thresholds, such as those outlined by FDA guidelines on licensure [22], the method for assessing these effectiveness rates needs to be carefully considered. Based on our observation of a consistent bias in HAI titers for EDTA plasma for influenza B, compared to SST methods, we recommend that the use of EDTA plasma be considered with extreme care, or avoided, for studies related to seroprevalence and seroconversion following vaccination.

The mechanism of the effect of sample collection method on differences in HAI and MN titers for influenza viruses remains to be determined. Previously it has been hypothesized that anticoagulants in plasma may affect HAI (and MN) titers by lowering titers overall. Indeed we have previously seen lower HAI titers for temporally matched sodium citrate plasma or heparin plasma compared to sera [21]. In the present study we found that RT sera, ACD plasma, heparin plasma, and 3.2% sodium citrate solution-plasma resulted in generally lower HAI and MN titer values (17/20 had a negative bias compared to SST), but statistically similar, compared to SST sera sample collection. EDTA plasma collection resulted in higher HAI titer values for influenza A and B, with the values being significantly higher for influenza B, compared to SST. However, EDTA plasma collection did not significantly change MN assay results compare to SST. This finding could be potentially due to more carry over of residual anticoagulants for HAI assays that is not seen for MN assays. Anticoagulants may bias towards higher titer results particularly at lower overall titers (as assessed by SST). EDTA is a chelating agent and could hypothetically influence the ability to detect antibody/antigen interactions. Indeed, EDTA treatment of serum has been shown to improve human leukocyte antigen detection [33]. Anticoagulants in plasma collection tubes may also introduce other interferences related to enzyme inhibitors, fibrinogen, and cations [34] that may influence HAI and MN titers. Variations could also occur for concentrations of anticoagulants present in tubes, perhaps particularly for EDTA and acid citrate dextrose. However, how EDTA presence may affect Type B influenza HAI titers in particular remains to be elucidated.

The present study has a number of limitation that need to be considered, including limitations related to the viruses used and limitations related to the choice of blood samples used. In the present study, we focused on three strains of influenza representing two type A (an H1N1 and an H3N2) and one type B virus, and representing strains capable of seasonal or pandemic influenza. We were unable to test one of the viruses (Type A H3N2) in both the HAI and MN assay for all collection methods. A limitation of this study is that we did not: (i) investigate additional types of influenza such as C or D; (ii) further investigate additional strains/lineages for influenza B; and (iii) further investigate additional subtypes/strains for influenza A based on hemagglutinin and neuraminidase subtypes. The potential differences in titer values for HAI and MN assays amongst different collection tube methods should, in particular, be assessed for additional A and B influenza viruses as these results would have broader implications for assessing seasonal influenza vaccines and assessing seroprevalence. Similarly, additional viral factors that may influence assay results could be investigated in future studies including: (i) comparing cell-derived versus egg-derived viral strains; and (ii) the effect of ether splitting influenza B viruses prior to conducting serological assays. As newer cell-based influenza vaccines become more common, properly assessing seroconversion rates will have implications for licensure. Therefore both egg-derived and cell-derived viruses should be assessed for influence upon different sample collection methods and serological results. An additional viral factor for consideration in future similar studies would be to assess the effect of ether splitting influenza B viruses prior to assessing serological results. This is important as ether splitting the virus prior to conducting the HAI assay might increase the dynamic range of the assay (and may result in higher titer values). This has implications for the present study as many of the blood samples collected had low HAI titer values (all below 320) but not MN titer values for influenza B, perhaps due to not conducting an ether split.

An additional limitation of this study is related to the choice of samples collected. In this study we utilized blood samples, collected six different ways, from thirty healthy donors previously immunized against influenza viruses. As such this study lacked seronegative controls. In the present study the closest to a seronegative control was sample #9, were all titer values for each virus and each assay for SST were < 40 and all additional titer values for each collection method were ≤ 80. Additionally, the spread of titer values could have ideally been greater. As a result of these two sample selection factor limitations it is more difficult to definitively interpret the findings of the present study. Future investigations into the effect of sample collection methods on serological assays should incorporate susceptible/seronegative controls, and should ideally include more samples with greater titer spread in order to better assess potential non-specific reactivity at lower antibody levels. Further investigation of serological blood collection methods, as well as additional factors that influence assay results, is warranted as we seek to standardize influenza serological assays and improve inter-laboratory cooperation globally in order to better interpret influenza serological assays, estimate influenza severity and attack rates, and inform on public health policy.

## Conclusions

Throughout this study we used the same standardized HAI and MN assay protocols for all matched samples, with the only difference being the blood collection method. Based on these results, and with proper consideration of the limitations of this study, we recommend that the SST sample collection method remain the gold standard for influenza serology, but that other methods could be used where convenient/necessary. We saw a high degree of agreement (93% or better) in HAI and MN titer values for matched samples against three different influenza viruses when using RT Sera, Hep plasma, and Cit plasma and comparing to SST values. Additionally, ACD plasma may also be used in place of or in addition to SST to measure HAI and MN titers if needed. The use of the EDTA plasma collection method should be considered with more caution. The mechanisms of the effect of anticoagulants on HAI and MN assays warrants further investigation in order to prioritize standardization of sample collection method for future efforts standardizing influenza serological assays.

## Additional file

Additional file 1: HAI and MN Titer Values. HAI and MN titer values for all influenza types for the thirty matched samples and for the six collection tube methods. (XLSX 27 kb)

## Abbreviations

Ab: Antibodies
ACD: Acid citrate dextrose
Cit Plasma: Citrate tubes
EDTA: Ethylenediaminetetraacetic acid
ELISA: Enzyme-linked immunosorbent assay
FDA: United States Food and Drug Administration
GMT: Geometric mean titer
HA: Hemagglutinin
HAI: Hemagglutination inhibition
Hep Plasma: Sodium heparin tubes
MDCK: Madin-Darby canine kidney
MN: Microneutralization
RBCs: Red blood cells
RDE: Receptor destroying enzyme
RT Sera: “red-top serum” tubes
SST: Serum separator tubes
TCID_50_: 50% tissue culture infective dose
TPCK: Tosyl phenylalanyl chloromethyl ketone
TREAT: t-test relative to a threshold
WHO: World Health Organization
